# Genome Sequences of 11 Conspecific *Streptomyces* sp. Strains

**DOI:** 10.1128/MRA.00863-19

**Published:** 2019-09-19

**Authors:** Abdoul-Razak Tidjani, Jean-Noël Lorenzi, Maxime Toussaint, Erwin van Dijk, Delphine Naquin, Olivier Lespinet, Cyril Bontemps, Pierre Leblond

**Affiliations:** a Université de Lorraine, INRA, DynAMic, Nancy, France; b Institute for Integrative Biology of the Cell (I2BC), CEA, CNRS, University Paris-Sud, University Paris-Saclay, Gif-sur-Yvette, France; Broad Institute

## Abstract

The genomes of 11 conspecific Streptomyces strains, i.e., from the same species and inhabiting the same ecological niche, were sequenced and assembled. This data set offers an ideal framework to assess the genome evolution of *Streptomyces* species in their ecological context.

## ANNOUNCEMENT

*Streptomyces* species are soil-dwelling bacteria that harbor large linear chromosomes (1). We report here the genome sequences of 11 sympatric *Streptomyces* strains belonging to the same species. In order to select conspecifics, we sampled soil grains at the cubic milliliter scale from a French forest (maximal distance of 8 cm from each other). After dissolution in sterile water and spreading of serial dilutions on *Streptomyces* isolation medium (SIM) (2), the 16S rRNA sequences of the strains were determined and analyzed using BLAST (3), and their phylogenetic relationships were characterized by multilocus sequence analysis (MLSA) using the Molecular Evolutionary Genetics Analysis version 7 (MEGA7) software (4). After growth in liquid Hickey-Tresner medium at 30°C for 30 h, DNA purification was performed using the salting-out method (5), followed by chloroform extraction. The targeted genes (rRNA gene and the MLSA genes) were amplified using universal (16S rRNA gene [6]) and specific (MLSA [7]) primers. The 11 strains selected showed identical 16S rRNA gene sequences and minimal MLSA divergence. These strains are related to Streptomyces olivochromogenes (strain DSM40451), with an average identity of 99.93% for the 16S rRNA gene sequences (8). A hybrid assembly using Oxford Nanopore technology for scaffolding and Illumina technology for sequence improvement was performed (Table 1). Base calling of these sequences was performed using the Oxford Nanopore base callers Albacore (v0.8.4 or v2.0.2) or Guppy (v0.3.0). Nanopore reads (minimum quality mean, 7) were generated on minION or gridION systems. When strains were multiplexed, Porechop (v0.2.4, using default settings) was used for demultiplexing (and adaptor trimming). The coverage ranged from 41× to 344×. The Illumina paired-end libraries were created using the Illumina Nextera kit, except for RLB1-8 and RLB1-9, for which sonication (Covaris) and adaptor ligation (Illumina TruSeq) were used instead. Paired reads were generated using a MiSeq reagent kit v3 (150 cycles) and the Genome Analyzer system (Illumina). The minimum read size was set to 10 bp, and adaptor trimming was performed using Cutadapt (v1.15, using default settings). The coverages of the paired-end reads (length, 75 to 300 bp) ranged from 58× to 320×. The hybrid assembly was performed using Unicycler (9) v0.4.2 or v0.4.3 (using default settings) to assemble 1 to 19 large contigs covering the whole genome of each strain, enabling the acquisition of each linear chromosome in one scaffold and the identification of extrachromosomal elements when present. One or two extrachromosomal linear or circular replicons were identified in 5 of the 11 strains by *in silico* prediction or pulsed-field gel electrophoresis experiments (10). The total genome sizes ranged from 11.76 to 12.45 Mb, positioning these strains among the largest bacterial genomes (Table 1).

**TABLE 1:** Genome features, sequencing statistics, and accession numbers of the 11 conspecific *Streptomyces* strains

Strain or replicon	Illumina sequencing information		Oxford Nanopore sequencing information						Replicon size (bp)^*c*^	Genome size (bp)	Total no. of CDS^*a*^	G+C content (%)	TIR^*b*^ (kb)	GenBank accession no.
	No. of reads (approx coverage [×])	SRA accession no.	No. of reads (approx coverage [×])	*N*_50_ of raw reads (kb)	Flow cell type(s)	Sequencing kit(s)	Base caller	SRA accession no.						
RLB1-8	15,381,622 (320)	SRR9661592	655,482 (150)	4.3	FAH18893 (9.5), FAH18988 (9.5), FAH24488 (9.5)	sqk-lsk308	albacore_2.0.2	SRR9710048	11,765,340	11,765,340	10,635	70.2	357	CP041650
RLB1-9	18,329,970 (115)	SRR9661591	88,694 (41)	7.6	FAF19789 (9.4)	sqk-lsk308	albacore_0.8.4	SRR9710047	11,940,408	12,200,709	10,838	70.2	311	CP041654
pRLB1-9.1									154,158^C^		175	69,0		CP041653
pRLB1-9.2									106,143^L^		111	68.7	24	CP041652
RLB3-5	3,196,108 (67)	SRR9661590	144,521 (56)	7.1	FAH24352 (9.5), FAH29240 (9.4)	sqk-lsk308, sqk-lsk108	albacore_2.0.2	SRR9710050	11,898,970	11,898,970	10,731	70.2	365	CP041651
RLB3-6	3,274,272 (68)	SRR9661589	299,155 (52)	4.6	FAF19789 (9.4)	sqk-lsk308	guppy_0.3.0	SRR9710049	12,338,263	12,448,281	11,255	70.1	587	CP041602
pRLB3-6.1									110,314^C^		101	70.6		CP041601
RLB3-17	3,976,622 (83)	SRR9661596	202,455 (50)	5.6	FAF19789 (9.4)	sqk-lsk308	guppy_0.3.0	SRR9710052	12,023,175	12,023,175	10,934	70.2	451	CP041610
S1A1-3	3,243,184 (68)	SRR9661595	198,567 51)	5.2	FAF19789 (9.4)	sqk-lsk308	guppy_0.3.0	SRR9710051	12,042,091	12,042,091	10,920	70.2	393	CP041611
S1A1-7	3,504,210 (73)	SRR9661594	533,299 (73)	2.8	FAF19789 (9.4)	sqk-lsk308	guppy_0.3.0	SRR9710054	11,713,151	12,005,504	10,580	70.3	513	CP041604
pS1A1-7.1									292,353^C^		252	69.7		CP041603
S1A1-8	3,191,318 (66)	SRR9661593	780,962 (53)	1.1	FAF19789 (9.4)	sqk-lsk308	guppy_0.3.0	SRR9710053	12,036,971	12,036,971	10,918	70.2	394	CP041612
S1D4-14	2,794,454 (58)	SRR9661598	2,066,754 (344)	3.2	FAF19789 (9.4)	sqk-lsk308	guppy_0.3.0	SRR9710056	11,723,487	11,934,498	10,591	70.2	369	CP041607
pS1D4-14.1									112,196^L^		118	68.7	0	CP041605
pS1D4-14.2									98,815^C^		138	69.1		CP041606
S1D4-20	3,327,172 (69)	SRR9661597	1,424,402 (255)	4.1	FAF19789 (9.4)	sqk-lsk308	guppy_0.3.0	SRR9710055	11,851,257	12,245,276	10,742	70.2	373	CP041609
pS1D4-20.1									394,019^L^		329	69.1	68	CP041608
S1D4-23	3,543,760 (74)	SRR9661599	400,348 (61)	3.5	FAF19789 (9.4)	sqk-lsk308	guppy_0.3.0	SRR9710057	12,057,712	12,057,712	10,971	70.2	421	CP041613

a As determined through automatic annotation by the NCBI Prokaryotic Genome Annotation Pipeline. CDS, coding sequences.

b TIR, terminal inverted repeat.

c ^L/C^, linear (^L^) or circular (^C^) replicon configuration, as predicted by the assembler and tested by pulsed-field gel electrophoresis (not shown).

### Data availability.

Genome sequences and raw sequence reads are available from GenBank and the NCBI Sequence Read Archive under the accession numbers shown in Table 1.
